# Granuloma Formation and Host Defense in Chronic *Mycobacterium tuberculosis* Infection Requires PYCARD/ASC but Not NLRP3 or Caspase-1

**DOI:** 10.1371/journal.pone.0012320

**Published:** 2010-08-20

**Authors:** Erin McElvania TeKippe, Irving C. Allen, Paul D. Hulseberg, Jonathan T. Sullivan, Jessica R. McCann, Matyas Sandor, Miriam Braunstein, Jenny P.-Y. Ting

**Affiliations:** 1 Department of Microbiology and Immunology, The University of North Carolina at Chapel Hill, Chapel Hill, North Carolina, United States of America; 2 Lineberger Comprehensive Cancer Center, The University of North Carolina at Chapel Hill, Chapel Hill, North Carolina, United States of America; 3 Department of Pathology and Laboratory Medicine and Cellular and Molecular Pathology Program, School of Medicine and Public Health, University of Wisconsin, Madison, Wisconsin, United States of America; University of Delhi, India

## Abstract

The NLR gene family mediates host immunity to various acute pathogenic stimuli, but its role in chronic infection is not known. This paper addressed the role of NLRP3 (NALP3), its adaptor protein PYCARD (ASC), and caspase-1 during infection with *Mycobacterium tuberculosis* (*Mtb*). *Mtb* infection of macrophages in culture induced IL-1β secretion, and this requires the inflammasome components PYCARD, caspase-1, and NLRP3. However, *in vivo Mtb* aerosol infection of *Nlrp3^−/−^*, *Casp-1^−/−^*, and WT mice showed no differences in pulmonary IL-1β production, bacterial burden, or long-term survival. In contrast, a significant role was observed for *Pycard* in host protection during chronic *Mtb* infection, as shown by an abrupt decrease in survival of *Pycard^−/−^* mice. Decreased survival of *Pycard^−/−^* animals was associated with defective granuloma formation. These data demonstrate that PYCARD exerts a novel inflammasome-independent role during chronic *Mtb* infection by containing the bacteria in granulomas.

## Introduction

*Mycobacterium tuberculosis* (*Mtb*) is the causative agent of tuberculosis, a disease affecting one-third of the world's population and killing 1.7 million people each year [1]. *Mtb* is spread by aerosol droplets from persons with active infection. Upon inhalation, *Mtb* travels to the lung where it infects resident alveolar macrophages [2]. This initial infection leads to an innate immune response, which includes stimulation of Toll-like receptors (TLRs) that recognize pathogens and are located on the plasma membrane and within endosomes of host cells. *Mtb* is specifically recognized by TLRs 2, 4, and 9 [3]. TLR activation upregulates transcription of proinflammatory cytokines interleukin-1β (IL-1β), tumor necrosis factor alpha (TNFα), and interleukin-6 (IL-6), which are essential for the recruitment of immune cells to the site of infection and controlling *Mtb* infection [4], [5], [6].

In addition to TLR recognition, a newly discovered class of intracellular danger sensing proteins, the nucleotide binding domain, leucine rich repeats-containing family proteins known as NLRs, sense pathogens and pathogen products in the cell cytoplasm [7]. With more than twenty members, the NLRs function in host protection against a broad range of danger signals. Several NLRs function in immunity through the formation of a mutli-protein complex known as an inflammasome [8]. When activated by a specific danger signal, the inflammasome forms and results in recruitment and processing of pro-caspase-1, which in turn processes IL-1β and IL-18 to their active forms for secretion from macrophages.

NLRP3 is the most characterized of all the NLR inflammasome-forming proteins due to its abundant expression in macrophages and activation in response to the largest number of identified stimuli. In humans, a gain of function mutation in *NLRP3* is associated with hyperinflammatory hereditary periodic fever syndromes with symptoms ranging from mild rash to severe joint swelling [9].

Apoptotic speck-like protein containing a CARD domain (ASC, also PYCARD) was originally identified as a speck-forming protein during apoptosis of HL-60 cells [10]. PYCARD has also been recognized as an adaptor protein that interacts with NLR proteins forming a protein inflammasome structure resulting in caspase-1 processing and subsequent IL-1β and IL-18 activation [11]. PYCARD has been identified as an adaptor protein for NLRP3 and NLRP1, and it is functionally required for the NAIP5 and NLRC4 inflammasomes [12]. Each inflammasome responds to a specific set of stimuli, although there is some redundancy between NLRs. Together, PYCARD, NLRP3, and caspase-1 are essential for macrophage IL-1β maturation in response to a broad range of stimuli including bacteria [13], [14] and viruses [15], [16], [17], [18]. In addition to these inflammatory functions, PYCARD association with NLR proteins is required for pyroptotic and pyronecrotic cell death [19], [20]. Although the host's innate immune response to *Mtb* infection is critical for the initial defense against bacteria, the adaptive immune response is ultimately required for containment of the infection in the chronic stage of disease. Adaptive immunity to *Mtb* infection is characterized by the appearance of antigen specific CD4^+^ T-cells that secrete interferon-gamma (IFN-γ), which is responsible for activating macrophages to kill intracellular bacteria [21]. CD8+ T-cells are also important for controlling bacteria during the chronic phase of *Mtb* infection [22]. Chronic *Mtb* infection is controlled by granuloma formation which contains, but does not eliminate bacteria [23]. Granulomas consist of a central core of *Mtb-*infected macrophages surrounded by successive waves of activated macrophages, giant multinucleated cells, epithelioid cells, lymphocytes, fibroblasts, and dendritic cells. A subset of granulomas undergoes central caseous necrosis due to proteinaceous dead cell mass. Mice form slightly different granulomas that do not form caseous necrotic centers, but otherwise possess the same cell types and similar granuloma organization to humans [24].

Pro-inflammatory cytokine regulation can be critical to long-term survival of *Mtb* infection. *In vivo* assessments of *IL-1α/β^−/−^*, *IL-1R^−/−^*, and *IL-18^−/−^* mice have shown that these cytokines play a role in limiting bacterial lung burden, regulating other cytokines, nitric oxide production, and forming organized granulomas [25], [26], [27]. Likewise, mice deficient in pro-inflammatory cytokines IL-6 and TNFα have increased mortality during *Mtb* infection [4], [5]. TNFα is important for granuloma formation and maintenance [28]. Therefore, these cytokines are not only important in the innate immune response to *Mtb*, but also in host defense during chronic *Mtb* infection.

High interest in the role of NLRs in host immunity has led to the study of inflammasome complexes in response to many pathogens. The research thus far has almost exclusively focused on the acute effects of pathogens and other NLR stimuli. Consequently, the *in vivo* role of the NLR inflammasome during chronic infection has not been studied. *Mtb* infection exemplifies a chronic infection of paramount public health interest. *Mtb* infects macrophages where it must thwart the host immune response to survive and replicate. Nlrp3 inflammasome proteins are expressed in macrophages; thus, we hypothesized that *Mtb* infection would induce inflammasome activation. Here we show that *Mtb* induced IL-1β secretion in human and mouse macrophages *in vitro* and this process was dependent on PYCARD, caspase-1, and NLRP3, but not NLCR4. *In vivo*, murine *Pycard* helps protect the host from death during chronic *Mtb* infection while the effects of *Casp-1* and *Nlrp3* were negligible. The inability of *Pycard^−/−^* mice to form organized granulomas and the reduced presence of lung dendritic cells indicates a breakdown in host defense against *Mtb*. Thus, we identify PYCARD as a critical protein involved in host response to *Mtb* infection in an inflammasome-independent manner.

## Results

### Virulent and attenuated *Mtb* require PYCARD, NLRP3, and caspase-1 for IL-1β secretion by cultured human cells

**Figure 1:**
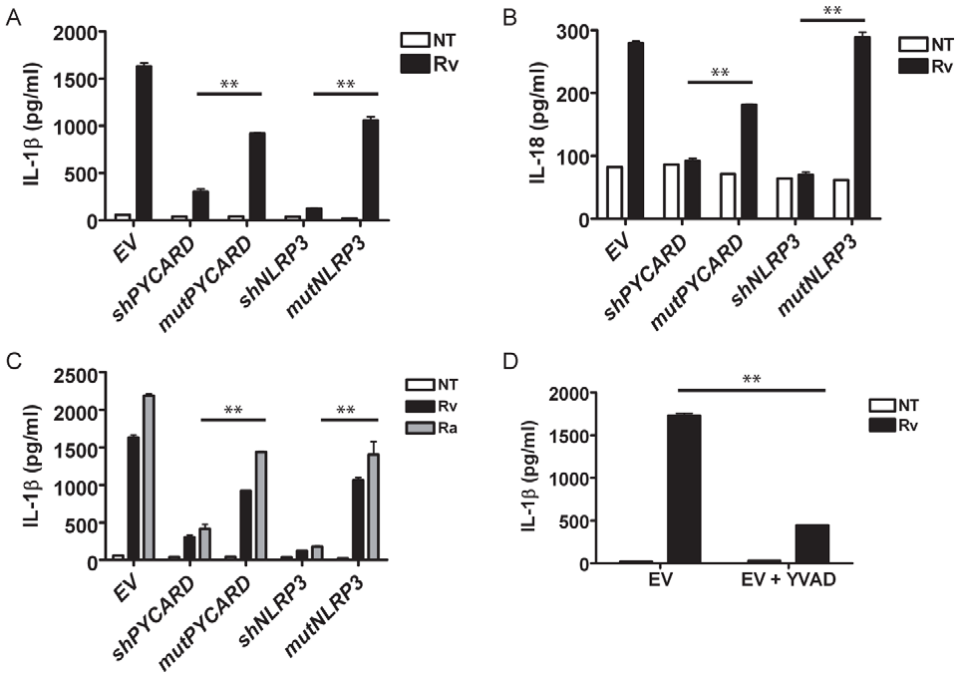
*Mtb* induced IL-1β in a human monocytic cell line required PYCARD and NLRP3. **A.** Virulent *Mtb* H37Rv induced IL-1β release was decreased in THP-1 cells stably transduced with PYCARD and NLRP3-specific shRNAs (shPYCARD and shNLRP3, respectively) but not in cells transduced with an empty vector (EV) or scrambled shRNAs (mut) or untreated (NT) cells. **B.** *Mtb-*induced IL-18 secretion was observed in EV and mut controls but not in shPYCARD and shNLRP3 cells. **C.** Attenuated *Mtb* H37Ra induced PYCARD and NLRP3-dependent IL-1β release. **D.** Caspase-1 specific inhibitor (YVAD-CHO, 100 µM) blocked *Mtb-*induced IL-1β. Error bars represent SD of a representative experiment. All experiments were repeated a minimum of three times. ** p<0.001.

During human *Mtb* infection, bacteria travel to the alveolar spaces where they infect and replicate inside macrophages. Secretion of IL-1β by macrophages requires pro-IL-1β processing by caspase-1. Caspase-1 interaction with several NLR forming inflammasomes results in caspase-1 processing, a prerequisite for IL-1β activation [12]. To determine if host detection of *Mtb* involves inflammasome activation, we used a used a panel of human monocytic THP-1 cell lines with reduced expression of inflammasome genes due to shRNA targeting sequences (**Table S1, Figure S1**). During infection with the virulent *Mtb* strain H37Rv, THP-1 cells with shPYCARD or shNLRP3 secreted significantly less IL-1β than their scrambled controls (**Figure 1A**). This indicates that *Mtb* activates the NLRP3 inflammasome. IL-18 is an inflammatory cytokine which induces cell-mediated immunity and causes T-cells to secrete IFN-γ [29]. Similar to IL-1β, it also requires caspase-1 cleavage for activation. IL-18 secretion from *Mtb-*infected THP-1 cells was dependent on PYCARD and NLRP3 (**Figure 1B**). These data demonstrated that both IL-1β and IL-18 were processed during *Mtb* infection and that each required the NLRP3 inflammasome formation for activation in the THP-1 human monocytic cell line.

As seen with H37Rv, the attenuated *Mtb* H37Ra strain induced IL-1β secretion from THP-1 cells in a PYCARD- and NLRP3-dependent manner. Induction of IL-1β by H37Ra was dependent on the NLRP3 inflammasome (**Figure 1C**). We consistently observed more IL-1β secretion in THP-1 cells infected with H37Ra, although the difference was not statistically significant. Thus, the NLRP3 inflammasome is activated by both virulent and attenuated *Mtb*. Chemically inhibiting caspase-1 with caspase-1-specific inhibitor Y-VAD significantly reduced IL-1β secreted by THP-1 cells, confirming the necessity of caspase-1 cleavage for IL-1β secretion (**Figure 1D**).

### Production of IL-1β in *Mtb* infected primary mouse macrophages is *Pycard, Nlrp3*, and *Casp-1* dependent

**Figure 2:**
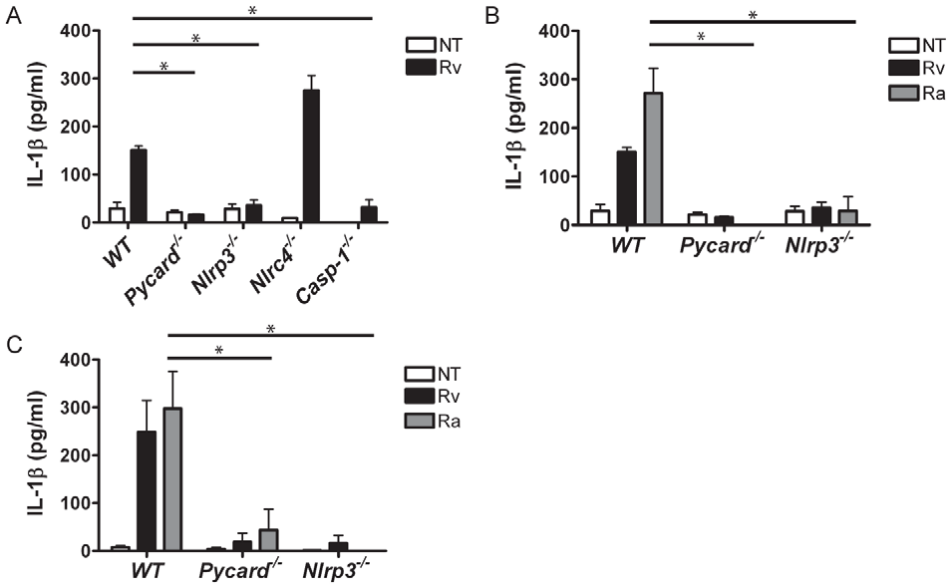
*Mtb* induced *Pycard, Nlrp3*, and *Casp-1* dependent IL-1β release in primary macrophages. **A.** *Pycard^−/−^*, *Nlrp3^−/−^*, and *Casp-1^−/−^* BMDM have decreased levels of IL-1β in response to *Mtb* H37Rv infection compared to WT or untreated (NT) BMDM. **B.** Attenuated *Mtb* H37Ra induced *Pycard* and *Nlrp3*-dependent IL-1β release. **C.** Thioglycolate elicited macrophages infected with *Mtb* H37Rv and *Mtb* H37Ra induced *Pycard* and *Nlrp3-*dependent IL-1β release. Error bars represent SD of a representative experiment. Each experiment was repeated a minimum of three times. * p<0.05.

To confirm our THP-1 data that the inflammasome is necessary for IL-1β processing, we infected primary mouse macrophages from gene depletion mice. We infected either bone-marrow derived (BMDM) or thioglycolated elicited macrophages with *Mtb*. *Mtb-*induced IL-1β secretion in wild type macrophages, confirming the results we obtained with THP-1 cells. *Mtb-*infected BMDM from *Pycard^−/−^*, *Nlrp3^−/−^*, and *Casp-1^−/−^* mice had significantly reduced IL-1β secretion, indicating that the Nlrp3-inflammasome is necessary for *Mtb-*induced IL-1β secretion in macrophages. *Nlrc4^−/−^* BMDM secreted IL-1β at the same or higher levels compared to wild type, indicating this inflammasome is not involved in the host response to *Mtb* (**Figure 2A**). This demonstrates congruent findings using THP-1 cells and primary mouse macrophages.

Induction of the NLRP3 inflammasome was not limited to virulent *Mtb* H37Rv infection, but also occurred with attenuated *Mtb* H37Ra (**Figure 2B**). Wild type BMDM infected with *Mtb* H37Ra secreted abundant amounts of IL-1β while *Pycard^−/−^* and *Nlrp3^−/−^* macrophages did not. Our findings were repeated in primary thioglycolate elicited macrophages. These highly activated macrophages had a significant reduction in IL-1β secretion in the absence of *Pycard* and *Nlrp3* (**Figure 2C**), reinforcing our data from human THP-1 cells and BMDM infected with virulent and attenuated *Mtb*. IL-18 secretion from both BMDM and thioglycolate elicited macrophages was below the level of detection for all samples tested. In cultured human monocytes and primary mouse macrophages *Mtb* induced IL-1β and IL-18 secretion in the absence of cell priming. This indicates that *Mtb* upregulates both transcription and processing of these proinflammatory cytokines *in vitro*. Taken together, our data show that the ability of *Mtb* to induce IL-1β secretion was PYCARD, NLRP3, and caspase-1 dependent in human THP-1 cells as well as primary mouse macrophages. In contrast, NLRC4 did not affect IL-1β production, indicating that *Mtb* induces a host response through the NLRP3, but not the NLRC4, inflammasome.

### *Pycard* but not *Nlrp3* protects the host against virulent *Mtb* infection

**Figure 3:**
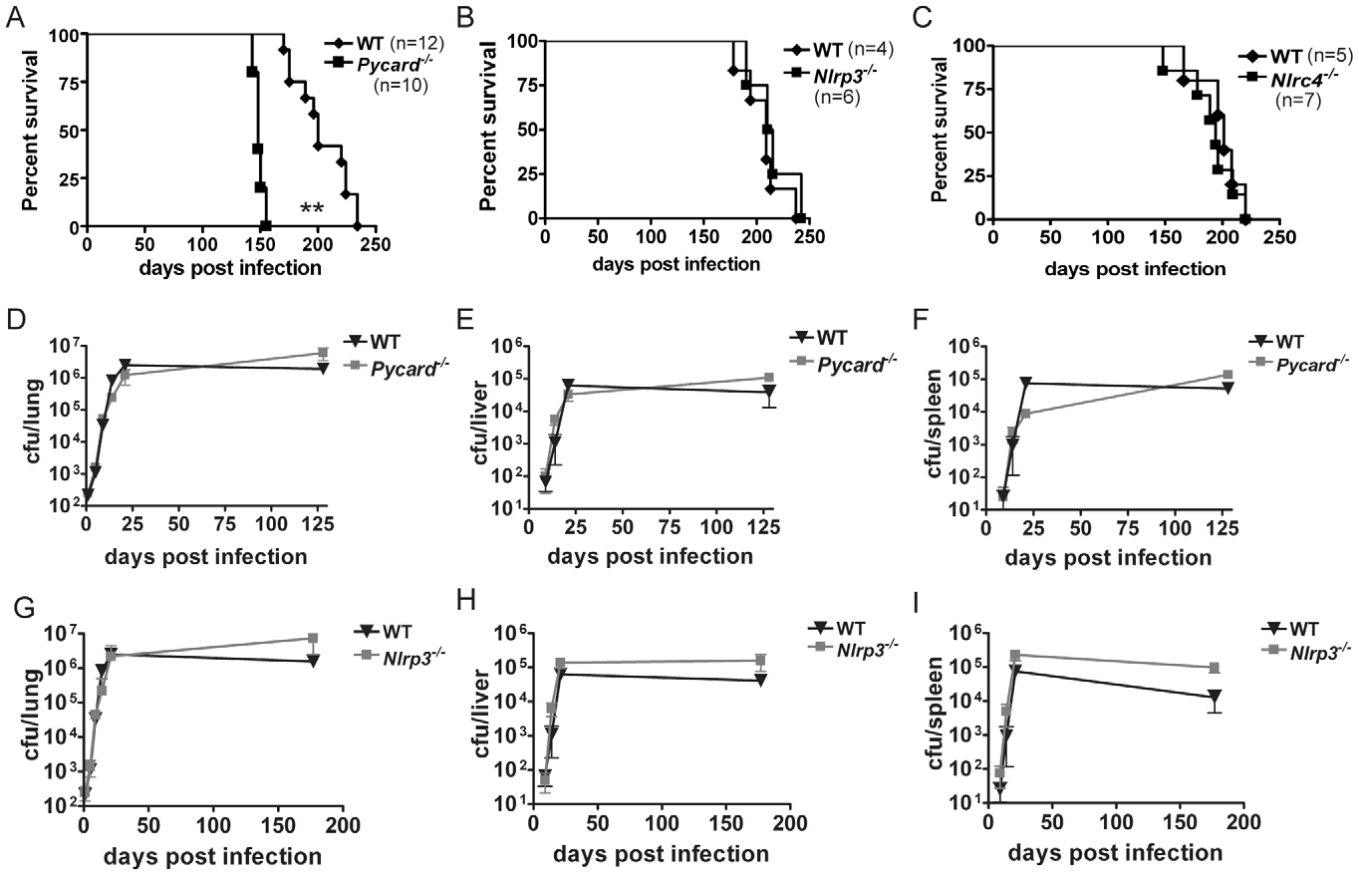
*Pycard^−/−^* mice were more susceptible to *Mtb* H37Rv aerosol infection, but bacterial burden was not increased. **A.** *Pycard^−/−^* mice infected with *Mtb* H37Rv die significantly earlier than age and sex matched wild type mice (**p<0.0001, log rank) **B–C.** No significant difference in survival was observed between *Nlrp3^−/−^* or *Nlrc4^−/−^* and WT mice after *Mtb* H37Rv infection. **D–I.** Bacterial organ burden. (**D,G**) Lungs, (**E,H**) liver, and (**F,I**) spleen of *Mtb-*infected *Pycard^−/−^* (**D–F**) and *Nlrp3^−/−^* (**G–I**) mice. Each gene-deletion mouse was individually controlled by concurrent infection of wild type mice. Error bars represent SD of a representative experiment. Organ burden data were obtained from two or more independent experiments with each containing at least three mice per genotype per time point.

Lack of IL-1β *in vivo* has been previously shown to be important for response to *Mtb* infection resulting in increased lung bacterial burden, differential regulation of cytokines, and defects in granuloma formation [6], [26]. Given our data from infecting cultured cells, we hypothesized that mice lacking the *Pycard* or *Nlrp3* would be unable to process IL-1β resulting in an inability to control *Mtb in vivo*. To test this hypothesis, wild type, *Pycard^−/−^*, and *Nlrp3^−/−^* animals were infected with aerosolized *Mtb* H37Rv. Each mouse received between 250–350 cfu per lung. During the first four-and-a-half months, survival of wild type and *Pycard^−/−^* mice was similar. However, *Pycard^−/−^* mice had a survival defect thereafter, and they died precipitously between 143 and 155 days post-infection with a mean survival of 148 days (**Figure 3A**). By comparison, wild type mice infected with *Mtb* had a mean survival of 200 days. These results were confirmed in a two additional experiments (not shown). Death of *Pycard^−/−^* mice during *Mtb* infection was not due to advanced age as we routinely keep *Pycard^−/−^* breeder pairs for up to a year with no unexplained deaths. This strong survival phenotype shows that *Pycard* is important for host defense against virulent *Mtb* infection *in vivo*.

PYCARD is an adaptor protein that is required for several NLR proteins to form inflammasomes. Since our *in vitro* data indicated NLRP3 was necessary for *Mtb* induction of IL-1β in macrophages, we also investigated survival of *Nlrp3^−/−^* mice during aerosolized *Mtb* infection. Although NLRP3 is important for IL-1β secretion from cultured macrophages, there was no difference in survival of *Nlrp3^−/−^* mice *in vivo* (**Figure 3B**). Overall, *Nlrp3^−/−^* mice had a mean survival of 209 days compared to 212 days for wild type mice. This data was also confirmed in a second experiment (not shown). In a separate experiment, we tested the ability of another closely related NLR gene, *Nlrc4*, to protect the host from *Mtb*. *Nlrc4^−/−^* mice had a similar survival profile to paired wild type controls, with a mean survival of 190 days compared to 198 days for wild type controls (**Figure 3C**). These data indicate that elimination of *Pycard*, but not *Nlrp3* nor *Nlrc4*, resulted in a reduced ability of the host to defend against *Mtb* infection.

### *Pycard* and *Nlrp3* do not significantly affect *Mtb* bacterial burden

Prior *in vivo* studies show that *Mtb* has two phases of infection [30]. In the first three weeks following aerosol infection, *Mtb* grows logarithmically in the lungs. After three weeks of infection, an effective Th1 adaptive immune response is established and the exponential growth of *Mtb* ends. *Mtb* bacterial burden in the lungs, spleen, and liver persist at the same level for the remainder of the infection [31]. We assessed bacterial burden in the lungs of *Pycard^−/−^* and *Nlrp3^−/−^* mice in two separate infections, with each compared to paired C57BL/6 controls. Animals lacking *Pycard* and *Nlrp3* have similar levels of bacteria in the lungs during the growth and persistence phases of *Mtb* infection compared to wild type (**Figure 3D, G**). The last time points were taken as *Pycard^−/−^* and *Nlrp3^−/−^* mice neared death. We saw no statistical difference between the bacterial burden of lungs from *Pycard^−/−^*, *Nlrp3^−/−^*, or wild type mice. The bacterial burden in the liver and spleen of the *Pycard^−/−^* and *Nlrp3^−/−^* mice was also similar to that found in wild type mice, indicating that *Pycard* and *Nlrp3* do not affect bacterial dissemination (**Figure 3E–F, H–I**). Thus, a change in bacterial burden and dissemination cannot explain the different survival rate of *Pycard^−/−^* compared to WT or *Nlrp3^−/−^* mice.

### Pro-inflammatory cytokines are produced at similar levels in the lungs of wild type, *Pycard^−/−^*, and *Nlrp3^−/−^* mice following *Mtb* H37Rv aerosol infection

**Figure 4:**
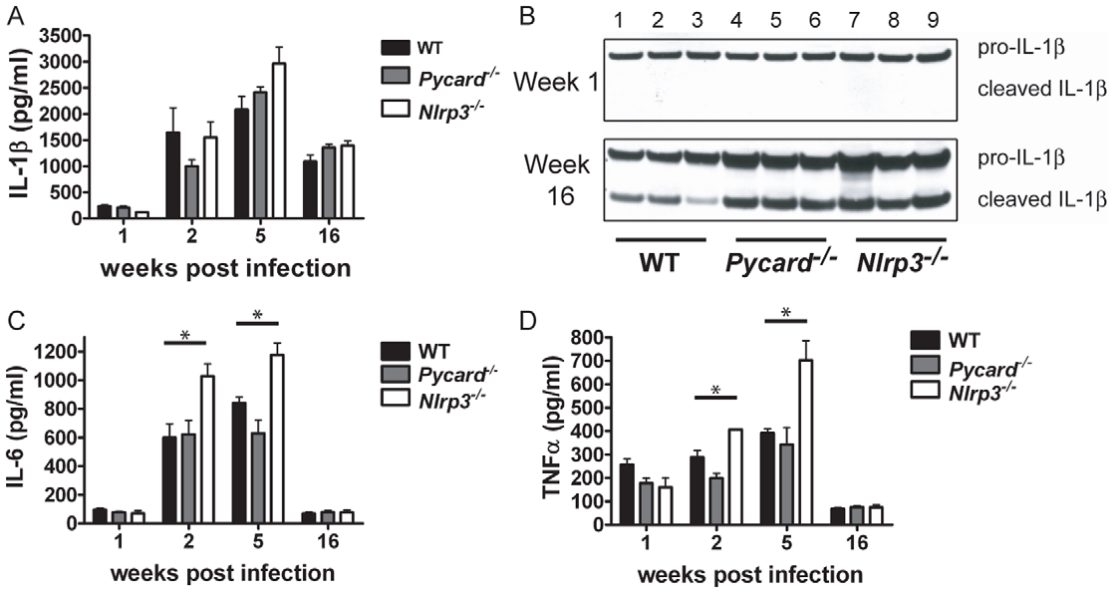
During *Mtb* infection wild-type, *Pycard^−/−^* and *Nlrp*3^−/−^ mice produced similar levels of mature IL-1β. Lung homogenates were assayed for proinflammatory cytokines. **A–B.** IL-1β was present in the lungs of *Pycard^−/−^* and *Nlrp*3^−/−^ mice by ELISA (**A**) and western blot (**B**) one week (**B, top panel**) and 16 weeks (**B, bottom panel**) post aerosol infection. Each number represents a different mouse. **C–D.** IL-6 (**C**) and TNFα (**D**) were modestly increased in *Nlrp*3^−/−^ lungs compared to *Pycard* ***^−/−^*** and WT. Cytokine measurements were taken from at least three mice per genotype per time point in two independent experiments. *p<0.05.

Our earlier studies led us to hypothesize that less IL-1β would be produced in the lungs of *Pycard^−/−^* and *Nlrp3^−/−^* mice compared to wild type because the gene deletion mice would not be able to cleave pro-IL-1β to its active form in response to *Mtb* infection. To test this, cytokines were measured from tissue free, homogenized lung extract by ELISA. Surprisingly, we found all three groups of animals produced IL-1β in similar amounts. IL-1β levels increased during the logarithmic growth phase of infection and were still high during the persistence phase of infection (**Figure 4A**). By week 16, levels of IL-1β decreased slightly. There was no significant reduction in IL-1β secretion between *Pycard^−/−^* and *Nrp3^−/−^* mice compared to wild type at any of the time points we measured. Because of the mechanical nature of lung homogenization, cells are broken open in the process which could result in release of both pro and cleaved IL-1β in the lung homogenate extracts. We performed IL-1β western blots in addition to ELISAs to ensure we were measuring the amount of cleaved IL-1β produced in the lungs of *Mtb*-infected animals *in vivo*. At one week post-infection, as expected, only pro-IL1β was present (**Figure 4B** **, top panel**). At 16 weeks post-infection, *Pycard^−/−^* and *Nlrp3^−/−^* animals produced comparable if not enhanced levels of cleaved IL-1β compared to wild type mice (**Figure 4B** **, bottom panel**). The amount of IL-1β was reproducible between mice and correlates with the ELISA data. These data show that differences in IL-1β production cannot explain differences in the survival rate of *Pycard^−/−^* compared to WT or *Nlrp3^−/−^* animals. We also measured pro-inflammatory cytokines IL-6 and TNFα. TNFα is of particular interest as it is important for granuloma formation and the control of infection in *Mtb*-infected animals and humans [5], [28]. IL-6 has also been shown to be an important host response against *Mtb* [4]. Furthermore, a previous report showed that human PYCARD is required for TNFα and IL-6 expression upon bacterial infection [32]. Both cytokines followed similar trends as IL-1β with total cytokine levels rising during early infection and then dropping off significantly by week 16 (**Figure 4C and D**). *Nlrp3^−/−^* mice had a modest but statistically significantly increase in IL-6 and TNF-α cytokines at 2 weeks (p = 0.026 and p = 0.028, respectively) and 5 weeks post-infection (p = 0.004, p = 0.001, respectively), but all differences were diminished by 16 weeks post-infection. There were no significant differences in lung cytokine levels between *Pycard^−/−^* and wild type mice at any time points analyzed. Proinflammatory cytokines in serum and bronchoalveolar lavage fluid were below the level of detection. These data indicate that reduced TNFα and IL-6 are not correlated with increased death in *Mtb*-infected *Pycard^−/−^* mice.

### Caspase-1 is not required for survival, bacterial containment, or the production of proinflammatory cytokines in *Mtb* infected mice

**Figure 5:**
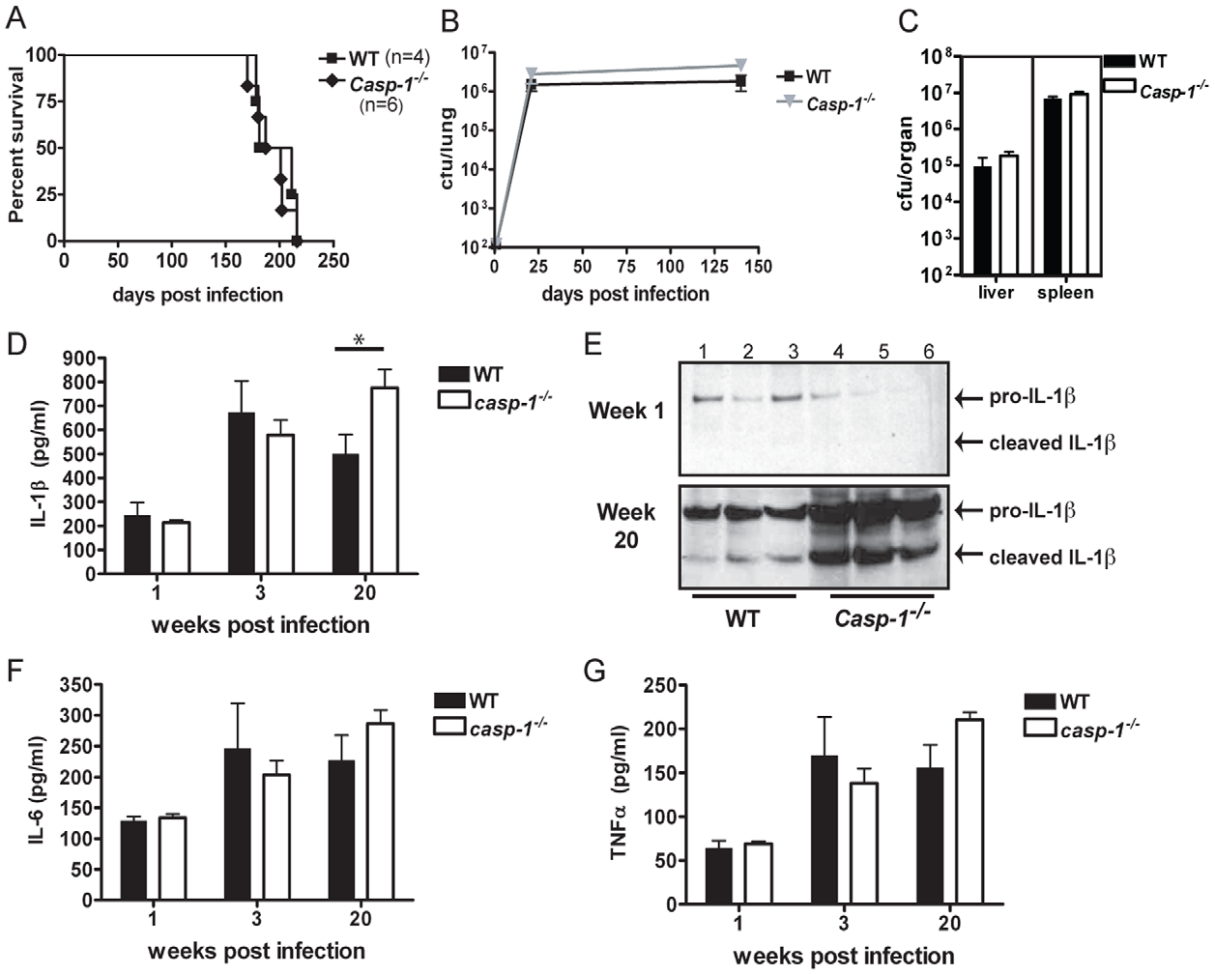
*Casp-1* was not protective during *Mtb* infection. **A.** *Casp-1* ***^−/−^*** mice infected with *Mtb* did not have a difference in survival compared to wild type mice. **B–C.** Bacterial burden in the lungs, liver, and spleen was comparable between *Casp-1^−/−^* and wild type mice. **D–G.** Lung homogenates were assayed for proinflammatory cytokines. **D.** *Casp-1* ***^−/−^*** lungs contained significantly more IL-1β than wild type lungs by ELISA (* p<0.05). **E.** IL-1β was confirmed by western blot of lung homogenates one week (**E, top panel**) and 20 weeks (**E, bottom panel**) post aerosol infection. **F–G.** There were no differences in the amount of IL-6 (**F**) or TNFα (**G**) present in *Casp-1^−/−^* and wild type lungs. Cytokine measurements were taken from at least three mice per genotype per time point in two independent experiments.

Caspase-1 recruits and cleaves IL-1β to its active form and is common to all NLR inflammasomes. A previous report has shown that at a low infectious unit of influenza virus, viral-induced host response is mediated by an *Nlrp3*-independent but *Pycard*/*Casp-1*-dependent inflammasome [15]. To assess if Caspase-1 and Pycard together mediate host protection in the context of inflammasome activation, we investigated the contribution of Caspase-1 during *Mtb* infection. Infection of *Casp-1^−/−^* mice with *Mtb* produced no difference in survival compared to wild type mice (**Figure 5A**). Measurement of bacterial burden showed that *Casp-1^−/−^* and wild type mice had similar levels of bacteria in the lungs over the course of *Mtb* infection (**Figure 5B**). *Casp-1^−/−^* and wild type mice also had similar bacterial burden in the liver and spleen, indicating that bacterial dissemination and growth in these organs are comparable between genotypes (**Figure 5C**). Overall, these data indicate that Caspase-1 does not play a prominent role in host protection during *Mtb* infection. Hence, this work describes a novel *Pycard-*dependent but *Casp-1-*independent form of host immunity.

Caspase-1 cleaves pro-IL-1β to its biologically active form. However, our earlier results demonstrated that cleaved IL-1β levels were similar in *Pycard^−/−^, Nlrp3^−/−^*, and *Nlrc4^−/−^* lungs. An analysis of *Casp-1^−/−^* mice show that rather than diminished IL-1β, they had increased lung IL-1β in their lungs compared to wild type as measured by ELISA (**Figure 5D**). We utilized western blots to measure both the pro- and cleaved forms of IL-1β from lung homogenate extracts collected one week and 20 weeks post infection. At one week post infection only low levels of pro-IL-1β were present in the *Casp-1^−/−^* and wild type lungs (**Figure 5E** **, top panel**). At 20 weeks post infection pro- and cleaved IL-1β were present in both *Casp-1^−/−^* and wild type mouse lungs with more found in the former (**Figure 5E** **, bottom panel**). These data demonstrate that *Casp-1^−/−^* mice have compensatory mechanisms of cleaving IL-1β in the absence of caspase-1. Levels of IL-6 and TNF-α also were unchanged between *Casp-1^−/−^* and wild type lungs (**Figure 5F–G**). Overall our *in vivo* characterization of the *Casp-1^−/−^* mouse reveals that Caspase-1 does not play a prominent role in host protection against *Mtb*. This supports the conclusion that protective host immunity to *Mtb* is independent of the inflammasome.

### *Pycard^−/−^* mice form fewer granulomas

**Figure 6:**
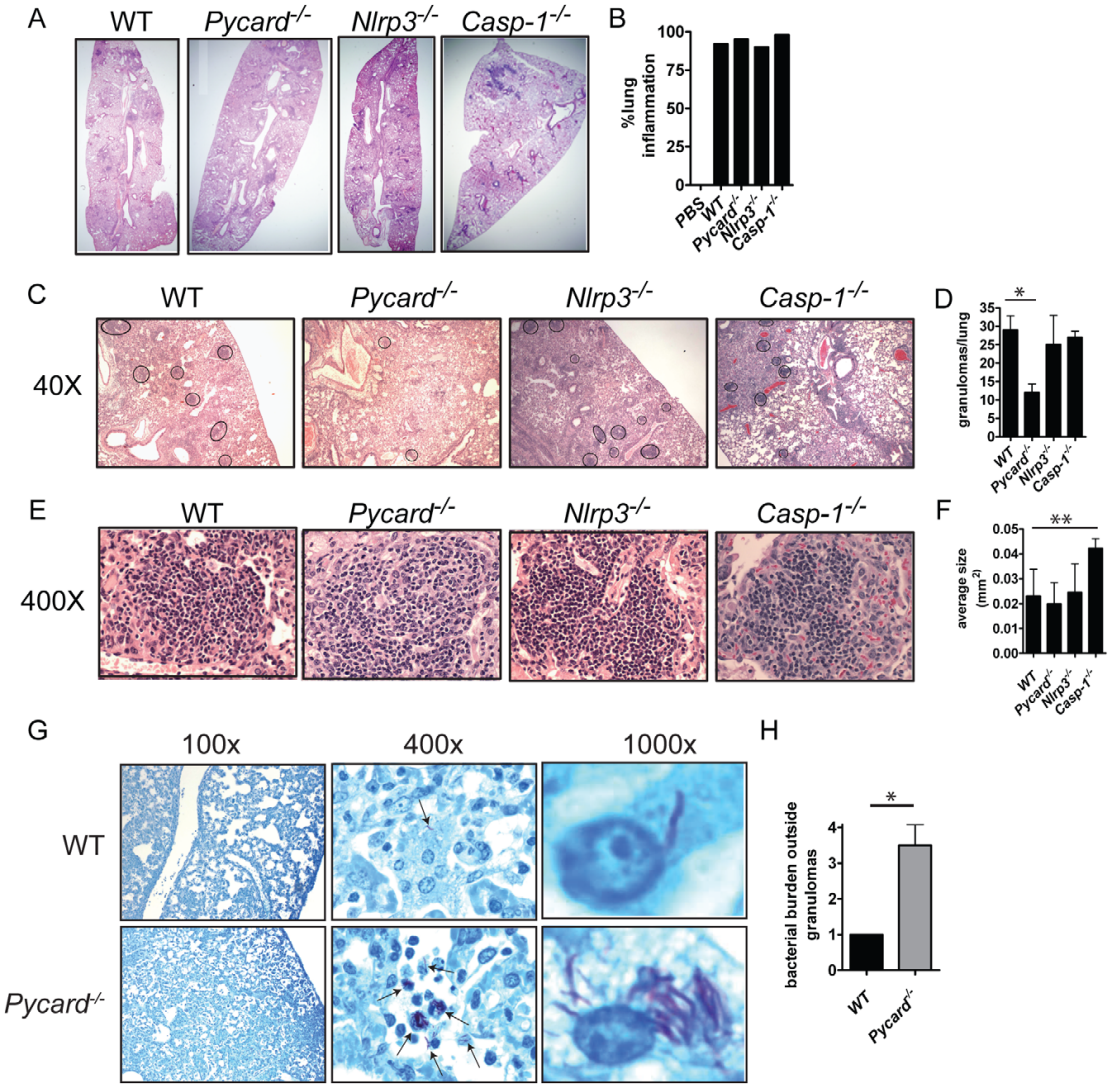
*Pycard^−/−^* mice had fewer lung granulomas and were unable to contain bacteria within granulomas during chronic *Mtb* infection. Histopathological analysis of lungs 16 weeks post *Mtb* infection. **A–B.** Whole lung images showed similar percentages of inflamed lung tissue in all genotypes of *Mtb-*infected mice. **C–D**. 40× images demonstrate that *Pycard^−/−^* mice form significantly fewer granulomas compared to wild type, *Nlrp3^−/−^*, and *Casp-1^−/−^* mice (* p<0.05). **E–F.** 400× images demonstrate wild type, *Pycard^−/−^*, and *Nlrp3^−/−^* mice form similarly sized granulomas while *Casp-1^−/−^* mice form significantly larger granulomas (**p<0.001). **G–H.** Images of acid-fast stained lung sections from non-granulomatous regions of *Mtb-*infected lung tissue were taken at 100× and 400×. *Pycard^−/−^* mice have bacterial located throughout the lung unlike wild type mice in which bacteria is mainly contained within granulomas (*p<0.01). To view bacteria within a single macrophage, 1000× images were cropped, and enlarged. Quantification was performed on sections from 4–8 mouse lungs per genotype from two independent experiments.

To further explore the mechanism by which *Pycard* enhances survival in chronically infected mice, we examined the histopathology of *Mtb-*infected mouse lungs. We examined granuloma structure because of its role in containing bacteria during chronic *Mtb* infection [23] and because *Pycard^−/−^* mice exhibit an abrupt drop in survival during the chronic phase of infection. We assessed lung inflammation 16 weeks post infection by measuring the amount of inflamed tissue from each mouse lung (see experimental procedures). Low magnification images showed that the alveolar spaces of wild type, *Pycard^−/−^*, *Nlrp3^−/−^*, and *Casp-1^−/−^* mice were obstructed by an influx of immune cells into the lung. We found the lungs of all our gene depletion and wild type mice to be highly inflamed, ranging from 90–98 percent of the total lung tissue (**Figure 6A–B**). In all infected mice, we observed dense areas of immune cells, indicative of granulomatous lesions. Strikingly, the *Pycard^−/−^* animals formed significantly fewer granulomas per lung compared to wild type, *Nlrp3^−/−^*, and *Casp-1^−/−^* lungs (**Figure 6C–D**). Examination under higher magnification showed that wild type and *Nlrp3^−/−^* mice formed similar sized granulomas. Hence, *Pycard* affected the formation of granulomas, but once they were formed, granuloma size was not affected by this gene (**Figure 6E–F**). *Casp-1^−/−^* mice formed larger granulomas, although this did not affect the outcome of infection. To further assess the lung granuloma defect in *Pycard^−/−^* mice, we stained mouse lung sections with Ziehl Neelsen stain to identify *Mtb*. Earlier data showed that the overall lung bacterial burden was similar in wild type and *Pycard^−/−^* mice by bacterial plating of homogenized lungs (**Figure 3F–G**). In contrast, analysis of acid fast staining revealed striking differences in bacterial localization in wild type and *Pycard^−/−^* lungs. Acid fast staining of wild type lungs shows very little *Mtb* located in non-granulomatous lung tissue. In contrast, *Pycard^−/−^* mice contain bacteria throughout the lung and have extremely high amounts of bacteria in tissue that is not associated with granulomas (**Figure 6G**). The amount of bacteria located outside of the granuloma was quantified by using a scale of 0–4 based on the amount of bacteria present in lung tissue that was not associated with granulomas (see experimental procedures) (**Figure 6H**). *Pycard^−/−^* mice form significantly fewer lung granulomas than wild type mice. The acid-fast staining data demonstrate that *Pycard^−/−^* animals were unable to contain bacteria within granulomas, shown by massive amounts of *Mtb* located in non-granulomatous lung tissue compared to wild type mice. Reduced granulomas in the lungs of *Pycard^−/−^* provide plausible mechanisms to explain the decreased lifespan observed in these animals during chronic *Mtb* infection.

## Discussion

In this report, we focus on the ability of virulent *Mtb* to stimulate inflammasome activation and the role of the inflammasome in host defense against *Mtb*. Prior studies have implicated the inflammasome in mycobacterium infection *in vitro*. Consistent with our data, one report showed that *M. marinum*, a mycobacterium that naturally infects fish and amphibians, activates IL-1β production in a PYCARD, NLRP3, and caspase-1-dependent manner *in vitro*, but no *in* vivo investigation was conducted [33]. Another report showed that *M. bovis* BCG, the vaccine strain of *Mtb*, can limit caspase-1 and IL-1β activation due to a putative protease Zmp1. In this latter study, however, there was no analysis of *Pycard, Nlrp3* or *Nlrc4* to directly implicate the inflammasome components [34]. A third report showed that *Mtb* infected THP-1 human monocyte cell line secrete IL-1β in a PYCARD and NLRP3-dependent process, but did not investigate the role of the inflammasome *in vivo* [35].

Our study showed that virulent *Mtb* H37Rv, along with the attenuated derivative *Mtb* H37Ra, induced NLRP3, PYCARD, and caspase-1-dependent inflammasome activation in a human macrophage cell line and in primary mouse macrophages. However, these *in vitro* results do not predict the outcome in mice. Most importantly, our study showed that *Pycard* is important for host protection during chronic infection with virulent *Mtb* infection *in vivo*, demonstrated by decreased survival of *Pycard^−/−^* mice compared to wild type controls. *Pycard* did not confer a survival advantage through the production of IL-1β. Rather it functions to prolong host survival through a novel inflammasome-independent role likely through proper granuloma formation during chronic *Mtb* infection. Surprisingly, neither *Nlrp3* nor *Casp-1* played a prominent role in host protection during *Mtb* infection despite its importance in culture. The inconsistencies between the *in vitro* and *in vivo* role of *Nlrp3* and *Casp-1* underscores the absolute necessity for *in vivo* validation in relating host immune genes to the outcome of microbial infections.

IL-1β cleavage is a hallmark of inflammasome activation, and we observed significant *Pycard, Nlrp3*, and *Casp-1*-dependent inflammasome activation during *in vitro* infection of cultured cells. However, in our *in vivo* studies, we observed mature IL-1β in the lungs of *Mtb* infected wild type, *Pycard^−/−^*, *Nlrp3^−/−^*, and *Casp-1^−/−^* mice. This, along with mouse survival data, indicates that Pycard host protection during *Mtb* infection *in vivo* is inflammasome independent. The similar survival profiles of *Nlrp3^−/−^* and *Casp-1^−/−^* mice compared to wild type mice may be due to compensatory mechanisms of IL-1β processing. It is important to note that the inflammasome is not the only host mechanism for cleaving pro-IL-1β to its active form. Through less well established, caspase-1-independent IL-1β cleavage can be carried out by several other host proteases including granzyme A, chymase, chymotrypsin, and matrix metalloproteinases as well as bacterial enzymes [36], [37], [38], [39]. Meanwhile, *Pycard^−/−^* mice lacked an additional mechanism to form or maintain granulomas thereby compromising their resistance to *Mtb* infection.

To investigate the mechanism further, we show that *Pycard^−/−^* mice have fewer lung granulomas compared to wild type, *Nlrp3^−/−^*, or *Casp-1^−/−^* mice, despite having the same amount of inflamed lung tissue. Acid-fast staining for *Mtb* localization within the lung demonstrated that *Pycard^−/−^* mice are defective in containing *Mtb* within granulomas, as shown by large amounts of bacteria found in non-granulomatous lung tissue. In comparison, infected wild type mice have almost no bacteria found outside of granulomas. Our data show that overall the lungs of *Pycard^−/−^* and wild type mice have the same amount of bacteria. However, the localization of *Mtb* within the lung is radically different. These data support a working hypothesis that Pycard affects proper cell accumulation during chronic *Mtb* infection and promotes proper granuloma formation.

Recently, another group implicated an inflammasome-independent role for Pycard in host protection against *Mtb in vivo* [40]. In contrast to our data, this paper found that *Casp-1* deficient mice had decreased survival during *Mtb* infection. This may be due to differences in the amount of bacteria delivered to the mice or mouse housing conditions. Nonetheless, both their and our papers come to the same conclusion: that Pycard has a role in host protection independent of Nlrp3.

In summary, this work demonstrates that PYCARD promotes survival during the late phase of *Mtb* infection independent of inflammasome activation. This result also highlights the importance of PYCARD in the process of granuloma formation during chronic *Mtb* infection. Considering the high rates of chronic *Mtb* infection worldwide and the increasing number of multi-drug resistant and extensively drug resistant tuberculosis cases, our study suggests that understanding the role of PYCARD during *Mtb* infection may lead to new and more effective therapies to treat chronic *Mtb* infection. In contrast, the lack of a role for NLRP3 and caspase-1 in the *in vivo* containment of *Mtb* is encouraging for the development of inhibitors for autoinflammatory disorders. These results suggest that anti-NLRP3 and caspase-1 strategies are not likely to cause the inadvertent activation of latent *Mtb* infection in patients.

## Methods

### Ethics statement

All studies were conducted in accordance with the National Institutes of Heath Guide for the Care and Use of Laboratory Animals and were approved by the Institutional Animal Care and Use Committee (IACUC) guidelines of the University of North Carolina at Chapel Hill (protocol #07-170 and 09-195).

### shRNA knockdown THP-1 cell lines

THP-1 cells were obtained from ATCC. Generation of shRNA knockdown and control vectors for PYCARD and NLRP3 have been described previously [13], [32]. All shRNA hairpins were confirmed by sequencing (**Table S1**). Lentiviral packaging and transduction of THP1 cells has been previously described [41]. Verification by RT-PCR indicated a significant reduction in PYCARD and NLRP3 expression (**Figure S1 A–B**). Functionally, shPYCARD and shNLRP3 had reduced IL-1β production in response to LPS (**Figure S1C**).

### Bacterial strains

*Mtb* H37Rv and H37Ra were obtained from the laboratory of William R. Jacobs, Jr. [42] and ATCC, respectively. Bacteria were grown to log phase in Middlebrook 7H9 broth (Difco) with 0.2% glycerol, 1× albumin dextrose saline, and 0.05% Tween 80. Inoculum was assessed by plating infection media on Middlebrook 7H10 agar plates supplemented with glycerol and ADS as described above. Colony forming units (cfu) were counted after 21 days of incubation.

### *Mtb* infection of cultured cells

Cell lines were cultured in RPMI (Gibco) with 10% FBS (Hyclone). Bone marrow derived macrophages were harvested from 6- to 8-week-old mouse femurs and cultured for 6 days in DMEM supplemented with L-glutamine, non-essential amino acids, 10% fetal bovine serum, and 20% L929 conditioned media. Thioglycolate elicited macrophages were obtained by peritoneal lavage 4 days after intraperitoneal injection with 3% thioglycolate and cultured in DMEM supplemented as above. All cells were infected under BSL3 conditions with *Mtb* H37Rv or H37Ra at an MOI of 10 and incubated for 8 hours at 37°C with 5% CO_2_. Cell-free supernatants were harvested, double filtered with 0.2 µ filters, and assayed for cytokines by ELISA and western blot.

### *Mtb* H37Rv aerosol infection of mice

Generation of *Pycard* ^−/−^, *Nlrp3* ^−/−^, *Casp-1^−/−^*, and *Nlrc4* ^−/−^ mice [43], [44], [45] have been described previously and were backcrossed onto C57BL/6 (Jackson Lab) background for a minimum of 9 generations. Mice were routinely genotyped. Female *Pycard^−/−^*, *Casp-1^−/−^ Nlrp3^−/−^*,and *Nlrc4^−/−^* mice and age matched C57BL/6 female controls were infected via aerosol as previously described [46]. Mice received 250–350 colony forming units per lung, determined by sacrificing a subset of mice 1d post infection as described by others [46]. Bacterial organ burden was quantified by plating serial dilutions of lung, liver, and spleen homogenates on 7H10 plates containing cycloheximide (1 µg/ml) and carbenicillin (50 µg/ml) to minimize contamination. Animal infections and organ harvests were carried out under BSL3 conditions.

### Cytokine determination

Cytokines were measured from infected cell supernatants with human or mouse BD OptEIA IL-1β, TNFα, and IL-6 ELISA Sets (BD Biosciences) and IL-18 ELISA (MBL International). *In vivo* cytokines were measured from lung homogenate extracts by centrifuging homogenized lung tissue to create a tissue-free supernatant. Amounts of pro- and cleaved IL-1β in lung homogenate extracts were determined by western blot. Immunoblots were probed with goat anti-mouse IL-1β primary antibody (R&D Biosystems). Bands were visualized by Super Signal Chemiluminescence (Pierce).

### Histopathology

Lungs were fixed in 10% buffered formalin and stained with H&E to evaluate airway inflammation and identify granulomas. The percent of inflamed lung tissue was determined by dividing the inflamed areas by the total lung area. Granuloma frequency was determined by counting the number of granulomas present in the total lung section. Granuloma size was measured by defining the granuloma borders for 26 lesions using Image J software and determining the average. Ziehl-Neelsen (ZN) staining was used to determine bacterial localization. The entire *Mtb* infected mouse lung was scored on a scale from 0–4: 0 = no bacteria found outside of granulomas, 1 = 1–5 bacteria found outside of granulomas, 2 = 6–20 bacteria found outside of granulomas, 3 = approximately half of each field contains 20-50 bacteria outside of granulomas, and 4 =  greater than half of each field contains greater than 50 bacteria outside of granulomas. H&E lung sections were blindly scored by E.M., I.C.A., M.S., and P.H. ZN stained lung sections were blindly scored by M.S. and P.H. For all histology quantification 4–8 mouse lungs per genotype were analyzed.

### Statistics

Data are presented as the means +/− standard deviation (SD) unless otherwise noted. Analysis Of Variance (ANOVA) followed by Tukey-Kramer HSD for multiple comparisons was performed on complex data sets. Statistical significance for single data points was assessed by the Student's two-tailed t-test. Survival curves were generated utilizing the product limit method of Kaplan and Meier and comparisons were made using the log rank test. In all cases, a p-value of less than 0.05 was considered statistically significant.

## Supporting Information

Table S1 Target sequences for shRNA THP-1 cell lines. Target sequences for PYCARD and NLRP3 shRNA hairpins used to construct stable knockdown THP-1 cell lines.
(0.03 MB DOC)

Figure S1 Validation of shRNA THP-1 cell lines. A–B. To ensure shRNA THP-1 cell lines had reduced gene expression we measured PYCARD (A) and NLRP3 (B) gene transcript by RT-PCR. C. Functional knockdown was shown by reduced IL-1β production in shPYCARD and shNLRP3 THP-1 cell lines after LPS stimulation. The THP cells used in this work have been cited in two other papers [1], [2]. These data are not included as new data but intended to be confirmatory of previous work. 1. Taxman DJ, Zhang J, Champagne C, Bergstralh DT, Iocca HA, et al. (2006) Cutting edge: ASC mediates the induction of multiple cytokines by Porphyromonas gingivalis via caspase-1-dependent and -independent pathways. J Immunol 177: 4252–4256. 2. Willingham SB, Bergstralh DT, O'Connor W, Morrison AC, Taxman DJ, et al. (2007) Microbial pathogen-induced necrotic cell death mediated by the inflammasome components CIAS1/cryopyrin/NLRP3 and ASC. Cell Host Microbe 2: 147–159.
(0.18 MB DOC)
