# Supplementary material for: Granuloma Formation and Host Defense in Chronic Mycobacterium tuberculosis Infection Requires PYCARD/ASC but Not NLRP3 or Caspase-1
Source: PLoS One. 2010 Aug 20;5(8):e12320. doi: 10.1371/journal.pone.0012320 (PMC2924896; doi:10.1371/journal.pone.0012320)
Supplement: Table S1 — Target sequences for shRNA THP-1 cell lines. Target sequences for PYCARD and NLRP3 shRNA hairpins used to construct stable knockdown THP-1 cell lines. (0.03 MB DOC) [file pone.0012320.s001.doc]

**Table S1**

| Construct | Target sequence |
| --- | --- |
| shPYCARD | GCTCTTCAGTTTCACACCA |
| mutshPYCARD | GCTCTTCctggcCACACCA |
| shNLRP3 | GGATGAACCTGTTCCAAAA |
| mutshNLRP3 | GGATGAACgcaTTCCAAAA |
