# Supplementary material for: Granuloma Formation and Host Defense in Chronic Mycobacterium tuberculosis Infection Requires PYCARD/ASC but Not NLRP3 or Caspase-1
Source: PLoS One. 2010 Aug 20;5(8):e12320. doi: 10.1371/journal.pone.0012320 (PMC2924896; doi:10.1371/journal.pone.0012320)
Supplement: Supplementary file 2 [file pone.0012320.s002.doc]

Figure S1

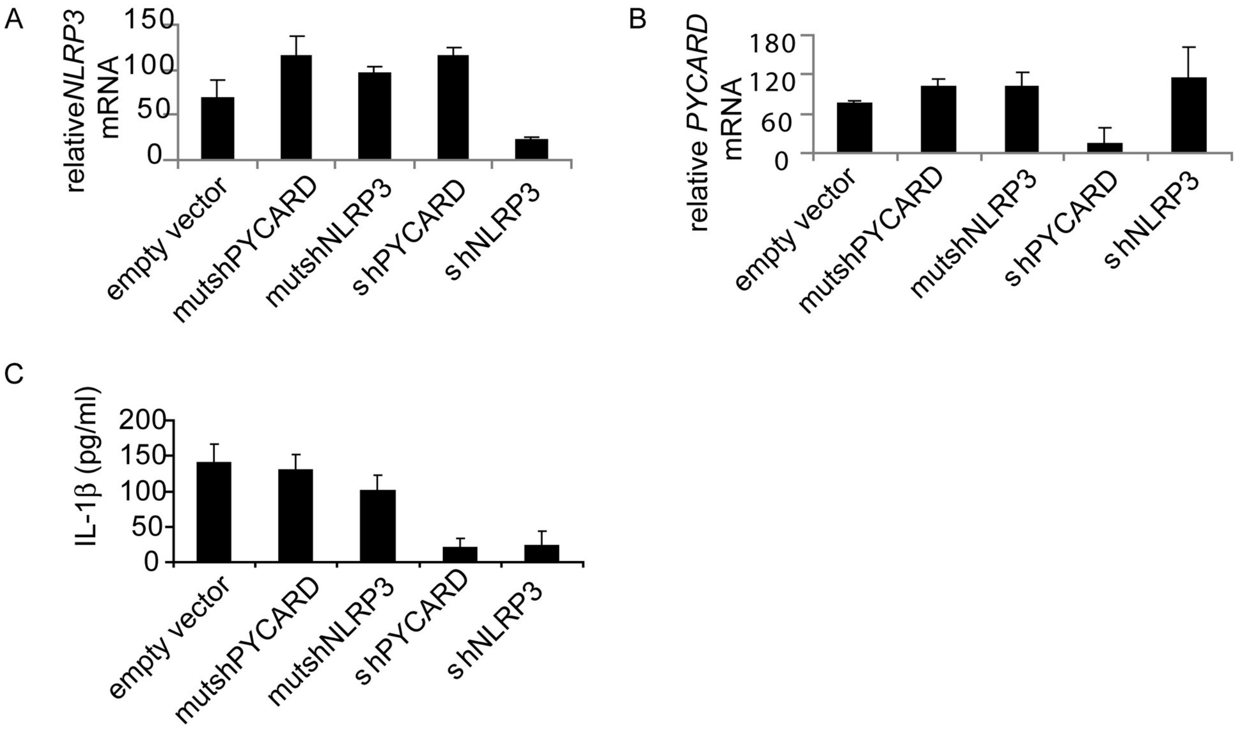
A
relative*NLRP3* mRNA
150
100
50
0
empty vector
mutshPYCARD
mutshNLRP3
shPYCARD
shNLRP3
B
relative *PYCARD* mRNA
180
120
60
0
empty vector
mutshPYCARD
mutshNLRP3
shPYCARD
shNLRP3
C
IL-1β (pg/ml)
200
150
100
50
0
empty vector
mutshPYCARD
mutshNLRP3
shPYCARD
shNLRP3
